# Prevalence of *Campylobacter jejuni* and *Campylobacter coli* in raw milk and some dairy products

**DOI:** 10.14202/vetworld.2016.1147-1151

**Published:** 2016-10-26

**Authors:** Mona A. El-Zamkan, Karima G. Abdel Hameed

**Affiliations:** Department of Food Hygiene and Control, Faculty of Veterinary Medicine, South Valley University, Qena 83523, Egypt

**Keywords:** *Campylobacter coli*, *Campylobacter jejuni*, dairy products, multiplex polymerase chain reaction, raw milk

## Abstract

**Aim:**

This study was accomplished to test raw milk and certain dairy products sold in local markets of Qena, Egypt, for the presence of *Campylobacter coli* and *Campylobacter jejuni*.

**Materials and Methods:**

A total of 150 samples of raw milk, kareish cheese, and yoghurt (50 samples each) were subjected first to enrichment in Bolton broth at 42°C for 2 days under a microaerobic condition, subsequently campylobacter blood free selective agar plates were cultured and incubated in the same condition of the broth. Based on the morphological and biochemical themes of the growing colonies, it was further classified into *Campylobacter* spp. The identified isolates were later affirmed by polymerase chain reaction using primers that were designed to locate *hip*O genes in *C. jejuni* and *gly*A in *C. coli*.

**Results:**

Of the total 150 examined samples of raw milk and soft cheese samples; 37 (24.6%) samples were contaminated with *Campylobacter* spp. *C. jejuni* was dominating in this study in 20%, 14%, and 8% of the examined raw milk, kareish cheese, and yoghurt samples, respectively. No sample harbored *C. coli*.

**Conclusion:**

*Campylobacter* spp. could be detected in 24.6% of the investigated samples. *C. jejuni* isolated from 14% of the total tested samples, while *C. coli* could not be detected from the examined samples. *Campylobacter* spp. is rampant in the areas of poor hygienic conditions making products made from raw milk of public health hazard.

## Introduction

Campylobacteriosis is a massed description for zoonotic diseases that caused by the bacterial genus *Campylobacter* which is accounted as a leading human food-borne pathogen and it is currently considered to be the main cause of bacterial gastroenteritis worldwide [1,2]. *Campylobacter* spp. initiated 7.5 million disability-adjusted life years in the study carried out by the Global Burden of Disease in 2010, it overtopped *Shigella* (7.1 million) and enterotoxigenic *Escherichia coli* (6.9 million) [3].

About 20 species are members of the *Campylobacter* genus, of these; *Campylobacter jejuni* and *Campylobacter coli* are responsible for most of the infections caused by this bacterium [4,5]. *Campylobacter* spp., mainly *C. jejuni* and *C. coli* induce enteric diseases that vary from a watery, nonbloody, non-inflammatory diarrhea to a severe inflammatory diarrhea with abdominal pain, fever, and malaise [5]. However, Guillain-Barré syndrome (GBS), which is a serious neurological disease with symptoms that include flaccid paralysis, Reiter’s syndrome or reactive arthritis may appear as serious postinfection sequelae [6-9]. The *Campylobacter* infection’s epidemiology in developed countries is significantly different to that in the developing world. In developing countries, *Campylobacter* enteritis has no preference for seasonality; in contrast, campylobacteriosis epidemics occur in summer and autumn in developed countries [1,10].

A number of transmission means have been blamed to the transmission of *Campylobacter* spp. to human, including consumption or handling of food as raw or underdone poultry or meat, raw milk and milk products [11]. Dairy products are predetermined as the main source of *Campylobacter* infection to human, as it ranked the first among food associated with Campylobacteriosis outbreaks [12,13]. This study aimed to explore the incidence of *Campylobacter* spp. in some dairy products with special concentration on *C. jejuni* and *C. coli* as a pathogen of major public health importance.

## Materials and Methods

### Ethical approval

Ethical approval is not required to pursue this type of study.

### Design of study

This study was conducted within September 2014-February 2015 in the Department of Food Hygiene and Control, Faculty of Veterinary Medicine, South Valley University, Qena, Egypt.

### Samples collection

A total of 150 samples of raw milk, kareish cheese, and yoghurt (50 samples each) were collected from local markets and street vendors in Qena city, Egypt. These samples were transferred to laboratory directly to be examined for the presence of *C. jejuni* and *C. coli*.

### Isolation of *Campylobacter* spp. from samples

The preparation of the samples and isolation of *Campylobacter* spp. from the examined samples was done according to FDA [14]. The pH of the samples was adjusted to 7.5±0.2, and then centrifugation of 50 g portion at 20,000 ×*g* for 40 min was attained. Supernatant was discarded and pellets were dissolved in 10 ml Bolton broth (supplemented with Bolton broth Selective Supplement and Laked Horse Blood, Oxoid) and then was transmitted to 90 ml enrichment broth and incubated at 42°C for 48 h in an anaerobic jar containing a gas generating Kit (Oxoid). The Campylobacter blood free selective agar (mCCDA-Preston, Oxoid) which was supplemented with CCDA selective supplement (Oxoid), were then streaked with a loopful of each enrichment broth, and subsequently, incubated at 42°C for 48 h under microaerobic condition. From 2 to 3 presumptive *Campylobacter* colonies were purified on Columbia blood agar (containing 7% defibrinated sheep blood)without supplement. About 100 *Campylobacter* isolates were submitted to Gram-stain, oxidase, catalase, inability to grow aerobically at 25°C, hippurate hydrolysis and resistance to naladixic acid and cephalothin to exclude *Campylobacter* spp. except *C. jejuni* and *C. coli*.

### Identification of *C. jejuni* and *C. coli* using multiplex polymerase chain reaction (mPCR)

From the biochemically confirmed *Campylobacter* isolates, 9 strains were selected to be submitted to PCR. From the tested strains, there were 4 suspected strains (one strain was giving a light grayish color in hippurate hydrolysis test, and the other 3 strains were suspected to be sensitive to nalidixic acid).

### DNA extraction

DNA extraction from isolates was operated using the QIAamp DNA Mini kit (Qiagen, Germany, GmbH) with remodeling of the manufacturer’s recommendations. In brief, 200 µl of the sample suspension was incubated with 10 µl of proteinase K and 200 µl of lysis buffer at 56°C for 10 min. Following the incubation, 200 µl of 100% ethanol was added to the lysate. The sample was thereafter washed and centrifuged according to the manufacturer’s recommendations. Nucleic acid was eluted with 100 µl of elution buffer afforded with the kit.

### Multiplex Polymerase Chain Reaction (mPCR)

mPCR was used to confirm *Campylobacter* isolates according to Wang *et al*. [15]. Primers used were supplied from Metabion (Germany). The used primers were intended to identify *hip*O genes in *C. jejuni* and *gly*A in *C.coli*. The primer sequences used are presented in Table-1.

**Table-1:** Sequences of the oligonucleotide primers.

Target agent	Target gene	Primers sequences (5′-3′)	Amplified segment (bp)	Reference
*C. jejuni*	*hipO*	ACTTCTTTATTGCTTGCTGC	126	Wang *et al*., 2002
		GCCACAACAAGTAAAGAAGC		
*C. coli*	*glyA*	GTAAAACCAAAGCTTATCGTG	323	
		TCCAGCAATGTGTGCAATG		

*C. coli=Campylobacter coli, C. jejuni=Campylobacter jejuni*.

PCR amplification and analysis of the PCR products.

The PCR mixture reaction (50 µl) consisted of 25 µl of EmeraldAmp Max PCR Master Mix (Takara, Japan), 1 µl of each primer of 20 pmol concentrations, 9 µl of water, and 12 µl of DNA template. Amplification of DNA was accomplished with 35 cycles of the following: Primary denaturation at 94°C for 10 min, annealing at 55°C for 30 s and extension at 72°C for 30 s with a final extension time of 72°C for 7 min (Table-1) in an Applied Biosystem 2720 thermal cycler. The products of PCR were separated by electrophoresis on 1.5% agarose gel (Applichem, Germany, GmbH) in 1 x TBE buffer at room temperature using gradients of 5 V/cm. For gel analysis, 30 µl of the products were loaded in each gel slot. A 100 bp DNA Ladder (Qiagen, Germany, GmbH) was used to determine the fragment sizes. The gel was photographed by a gel documentation system (Alpha Innotech, Biometra), and the data were analyzed through computer software.

## Results and Discussion

Results illustrated in Table-2 revealed that *Campylobacter* spp. were detected in 24.6% of the examined samples. No *C. coli* could be recovered from the samples, while *C. jejuni* could be isolated from 20% 14%, and 8% of raw milk, kareish cheese, and yoghurt samples, respectively. *C. jejuni* is now thought to be a major promoting agent of GBS and it is associated with several pathogenic profiles of GBS, axonal subtypes following the contagion may be more severe [16]. The infective dosage of *C. jejuni* is considered to be small, as human feeding studies submit that about 400-500 bacteria may produce illness in some persons, while in others, greater numbers are required [17].

**Table-2:** Incidence of *Campylobacter* spp. in the examined samples.

Samples	Number samples	No. (%)			

		*Campylobacter* spp.	*C. jejuni*	*C. coli*	Other *Campylobacter* spp.
Raw milk	50	11 (22)	10 (20)	0 (0)	1 (2)
Kareish cheese	50	17 (34)	7 (14)	0 (0)	10 (20)
Yogurt	50	9 (18)	4 (8)	0 (0)	5 (10)
Total	150	37 (24.6)	21 (14)	0 (0)	16 (10.6)

*C. coli=Campylobacter coli, C. jejuni=Campylobacter jejuni*.

The presence of *Campylobacter* spp. in raw milk may be contributed to contamination during milking process from the farm environment through feces [18], or after milking due to poor hygienic conditions during storage and handling of milk plus the major role played by workers in accelerating the incidence of the *Campylobacter* through cross contamination. Lower results recorded by Barakat *et al*. [19] who isolated *C. jejuni* from 4.4% of the investigated samples, while Yang *et al*. [17] obtained higher results as they could isolate *C. jejuni* from 26% of the examined raw milk samples. On the contrary, Muehlherr *et al*. [20] and Haghi *et al*. [21] could not isolate *C. jejuni* from milk samples and Gergs [22] isolated *C. coli* from 3% of the samples. Kareish cheese is one of the soft cheeses that are made from raw cow’s or buffaloes’ milk in farmers’ houses, so raw milk is a potential source of kareish cheeses contamination [23,24]. The usage of raw milk in the manufacturing of kareish cheese and the unhygienic conditions of preparation, processing, handling, storage, and selling methods explains the existence of highest positive samples among kareish cheese samples. The incidence of *C. jejuni* in this study is higher than Barakat *et al*. [19], who could isolate *C. jejuni* in 6.7% of the samples, while El-Sharoud [25] could not detect *Campylobacter* spp. in the examined kareish cheese samples. Contrary to our results Mina and Thanaa [26] isolated *C.coli* from kareish cheese samples.

The lowest incidence of *Campylobacter* spp. was found in yoghurt and this may have resulted from the low pH which hinders survival and growth of *Campylobacter* spp. [25]. The obtained result is closely related to those obtained by Aygun and Pehlivanlar [27] who could isolate *C. jejuni* from 6% of the samples while Barakat *et al*. [19] detected *C. jejuni* in a higher percent of the examined samples (13.4%). Like El-Sharoud [25], no *C. coli* could be determined in the inspected samples.

Substandard hygienic and sanitary condition and the tight closeness to animals in developing countries all backup the easy and recurrent attainment of any enteric pathogen including *Campylobacter*. Campylobacteriosis is deeply endemic in developing countries [10]. The major provenances of human infections are environmental pollution and foods. The results showed in this study display raw milk and dairy products as a mean of *Campylobacter* transmission.

Nowadays, in developing countries, as rule raw milk is boiled before being fed to babies, children, and other family members to protect them from fatal milk-borne infections, but still, there is a potential threat from consumption artisanal products made from raw milk. Taylor *et al*. [13] notified that *Campylobacter* outbreaks are much associated with contaminated dairy products as they found that dairy products were implicated in 65 (29%) out of 225 *Campylobacter* initiated foodborne outbreak in the US. Campylobacteriosis is a pediatric disease in developing countries as it has been stated that 60,000 per 100,000 children below 5 years of age are distressed by *Campylobacter* infections. In general, developing countries including Egypt do not have internal superintending recording system for *Campylobacter* foodborne outbreaks; therefore, incidence values expressed in the form of the number of patient cases for a population do not exist. Most evaluations of incidence in developing countries are collected from laboratory-based surveillance of pathogens responsible for diarrhea [28].

**Figure-1:**
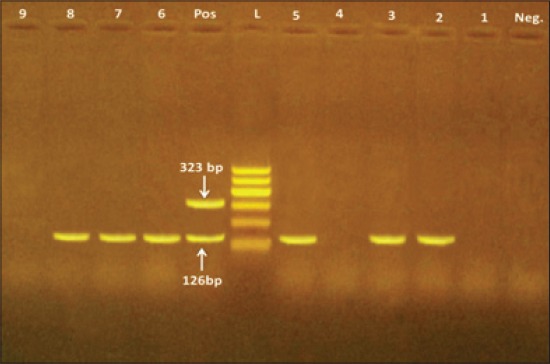
Multiplex-polymerase chain reaction of *Campylobacter jejuni* and *Campylobacter coli* strains isolates from raw milk, Kareish cheese and yoghurt samples. Lane (POS): Positive control. Lane (Neg): Negative control. Lane (L): 100 bp ladder as DNA marker. Lanes 2, 3, 5, 6, 7, 8 are positive for *C. jejuni* only. Lanes 1, 4, 9 are negative for both *C. jejuni* and *C. coli*. Lane 2: The strain gave a weak hippurate reaction.

The isolate that gave the weak reaction of the hippurate hydrolysis was confirmed to be *C. jejuni* using multiple PCR (Figure-1). That weak reaction, caused by that *C. jejuni* strain, may be resulted from using low bacterial concentration in the test. Nakari *et al*. [29] found that 32% of the 145 strains that gave the negative reaction in the standardized hippurate test turned out to be *C.jejuni* by PCR and 9 of these strains were responsible for an outbreak. These situations proved that phenotypic tests should be reinforced by the molecular method for the authoritative recognition of *C. jejuni* and *C. coli*; hence making the epidemiological statistics on the infections caused by *Campylobacter* spp. is more authentic.

## Conclusion

*Campylobacter* spp. occurred in 24.6% of the examined samples. About 56.7% of the isolated strains were identified as *C. jejuni* and no *C. coli* could be detected in the samples using culture and PCR methods. The high incidence of *Campylobacter* spp. in this study could be contributed to the unhygienic condition applied during production, and storage and also to the warm weather which help the microorganism to grow and multiply and also it shows the need for the increase awareness of the farmers and the small producers for the hygienic precautions during production.

## Authors’ Contributions

MAE conceived, designed the study, drafted and revised the manuscript. KGAH and MAE collected and analyzed samples. Both authors read and approved the final manuscript.
